# ‘Is there value for us in agriculture?’ A case study of youth participation in agricultural value chains in KwaZulu-Natal, South Africa

**DOI:** 10.1080/23311932.2023.2280365

**Published:** 2023-11-16

**Authors:** Wendy Geza, Mjabuliseni Simon Cloapas Ngidi, Maxwell Mudhara, Rob Slotow, Tafadzwanashe Mabhaudhi

**Affiliations:** 1 Centre for Transformative Agricultural and Food Systems, School of Agricultural, Earth and Environmental Sciences, University of KwaZulu-Natal, Pietermaritzburg, South Africa; 2 African Centre of Food Security, School of Agriculture, Earth and Environmental Sciences, University of KwaZulu-Natal, Pietermaritzburg, South Africa; 3 School of Agricultural, Earth and Environmental Sciences, University of KwaZulu-Natal, Pietermaritzburg, South Africa; 4 Department of Agricultural Extension and Rural Resource Management, School of Agricultural, Earth and Environmental Sciences, University of KwaZulu-Natal, Pietermaritzburg, South Africa; 5 Oppenheimer Fellow in Functional Biodiversity, Centre for Functional Biodiversity, School of Life Sciences, University of KwaZulu-Natal, Pietermaritzburg, South Africa; 6 International Water Management Institute (IWMI), Southern Africa Office, Pretoria, South Africa

**Keywords:** agriculture, inclusion, empowerment, food systems, participation, partnership, rural development, youth, Development Studies, Rural Development, Sustainable Development

## Abstract

Developing countries in Africa face an unemployment crisis, with many unemployed youth. Agriculture has been identified as a strategic sector for employment creation targeted at youth, including those who may not have agriculture-related qualifications. However, various challenges limit the effectiveness of youth participation in the agriculture value chain. The study aimed to (i) determine youth awareness of agricultural value-chain opportunities, (ii) determine their perception of their role in the value chain, (iii) determine their perception of agricultural programs targeting youth and their opinions on who is responsible for attracting youth into agriculture and, (iv) to characterise the dynamics of youth empowerment in agriculture. Data was collected using focus group discussions and an online survey and analysed using SPSS and NVivo. Poor knowledge, low levels of awareness of agricultural value-chain activities and careers, and not meeting the minimum requirements for employment in processing and retail businesses were identified as key challenges. Most youth were interested in non-primary activities such as agro-processing, which are less labour-intensive and have a quicker return on investment than agricultural production. However, support services and programs for promoting youth participation in agriculture mainly focus on primary activities, signifying a mismatch between youth aspirations and current support. Understanding youth aspirations, perceptions and dynamics underpinning youth empowerment and participation in value chains is critical for promoting participation and formulating relevant and responsive policies. Additionally, improving access to information and building awareness of agricultural value chains is crucial in reducing barriers to entry. Policymakers should integrate agriculture and food systems knowledge into the primary education curriculum to promote youth awareness and evoke interest in agri-food system careers at an early age.

## 1 Introduction

Agriculture and agro-processing are recognised as key areas that can boost regional trade and investments, promote structural transformation, create jobs, and eradicate hunger and poverty (Christiaensen et al., 2021; Das Nair & Landani, 2020; Schaffnit-Chatterjee et al., 2014). Agricultural value chains provide an additional opportunity for identifying possible remunerative income-earning opportunities for poor households and Africa’s growing youth population (Christiaensen et al., 2021; Haggblade et al., 2012). According to Dedehouanou et al. (2019), Africa, as a continent, is more specialised in agriculture than the rest of the world, primarily competitive in terms of price in the value chains of cotton, tea, sugar, sesame seeds, and cocoa. Africa’s regional participation in global value chains is driven by Southern and North Africa, which account for most of the continent’s total value chain trade—78%. West Africa accounts for only 14%, East Africa for 5%, and Central Africa for 3% (Conde et al., 2015). However, the current export structure of African countries demonstrates a lack of progress along the value chains. Of the US$ 62 billion in agricultural products Africa exported in 2017, only US$ 12 billion were classified as processed goods. While total exports increased between 2005 and 2017, the relative role of processed and unprocessed products has not evolved (Dedehouanou et al., 2019). Therefore, developing localised value-add in the agri-sector in Africa remains critical, as it is an economic growth and job-creation mechanism.

Strengthening localised value chains can provide economic opportunities for local people to diversify their livelihoods, influence food and nutrition security and contribute to environmental sustainability (Kumar & Sharma, 2016; Leach et al., 2020). However, agricultural value chains in sub—Saharan Africa are still underdeveloped (Dedehouanou et al., 2019). Some of the critical obstacles due to structural shortcomings are; accessing land and agricultural inputs, lack of skills, knowledge and functioning markets, inadequate infrastructure, affordable agriculture-related credit and lack of risk management mechanisms (Das Nair & Landani, 2020; Schaffnit-Chatterjee et al., 2014). Also, additional challenges caused by the effects of the COVID-19 pandemic on agricultural production and value chain activities (Ilesanmi et al., 2021), the exclusion of women and youth in income-generating activities (Malapit et al., 2020; Pyburn et al., 2015), growing inequity and inclusion that does not provide valuable pathways out of poverty (German et al., 2020).

Youth are considered key drivers of economic and social transformation in Africa because they are young, productive, energetic, better able to adapt to new technologies, and more educated and resilient (Fox et al., 2016; Yami et al., 2019). Additionally, Africa is experiencing a youth bulge, and youth-inclusive investments will likely play a crucial role in unleashing the agricultural sector’s potential (Christiaensen et al., 2021). Moreover, food-related activities predominate in strategies for creating employment opportunities for the growing youth population (Brooks et al., 2013; Girard, 2017). The value chain approach has been used to guide and direct sustainable initiatives to stimulate productivity, entrepreneurship, and the growth of Small and Medium Enterprises (SMEs) in Africa. Some examples include the African Agribusiness and Agro-industries Development Initiative (FAO & UNIDO, 2010), the LIVES capacity development project in Ethiopia (Lemma et al., 2016), and the African Technology Development Forum Entrepreneurship Hub (AEH) agri-business supporting young people in Malawi, Zambia and Zimbabwe (Konde et al., 2017)

The evidence suggests that some factors influencing the low participation of youth in agricultural value-chains are linked to a lack of awareness of value-chain opportunities, lack of access to finance, lack of or limited access to land, lack of education and training, limited access to markets, negative perceptions and lack of aspirations, inability to adapt and use technology (Andersson Djurfeldt et al., 2019; Christiaensen et al., 2021; Osti et al., 2015; Rim & Nsanganira, 2019; Smidt, 2021). There is low youth participation in agricultural value chains due to a lack of awareness of value chain opportunities and a limited understanding of where youth see themselves participating in the value chain. It was hypothesised that understanding youth perceptions of value chain participation is critical to unlocking their involvement. Two comprehensive scoping reviews were conducted to review existing literature on youth participation in agriculture and determine the dynamics of youth employment and empowerment in agriculture (refer to Geza et al. (2021) and Geza et al. (2022)). This study was developed to address the gaps identified in knowledge based on the findings of the scoping reviews. Given this background, the study seeks to investigate solutions to the following research questions;

(a) What is youths’ awareness of value chain opportunities?
(b) What are the perceptions of their role in the value chain?
(c) What are their perceptions on agricultural programs targeting young people, and who should be responsible for attracting youth into agriculture?
(d) What characterises the dynamics of youth empowerment in agriculture and rural development from a stakeholder’s perspective?

The specific objectives were:

(a) To determine youth awareness of value-chain opportunities and
(b) To determine their perception of their role in the value chain.
(c) To determine their perception of agricultural programs targeting young people and their opinions on who is responsible for attracting youth into agriculture.
(d) To characterise the dynamics of youth empowerment in agriculture and rural development from a stakeholders’ perspective.

### 1.1 Definition of terms and concepts

Within the context of this study, the main key terms and concepts used are defined as follows:

*Empowerment:* describes the process of nurturing youths’ ability to negotiate, take control, influence, and hold accountable institutions and procedures that affect their lives (Nnadi et al., 2012). It maximises young people’s chances to contribute to their society’s economic, social, and cultural advancement while engaging in capacity-building activities (Segun-Alalade et al., 2021). Therefore, empowered people have greater agency to bring about change. In addition, they are self-reliant and better equipped to tackle challenges (Mwesigwa & Mubangizi, 2019).

*Food systems:* include actors and various role players along the food value chain—from input supply to food consumption and disposal. It also includes the policy-enabling environment, institutions, and cultural norms around food and associated outputs—nutritional outcomes and socio-economic and environmental impacts (Fan & Swinnen, 2020).

*Participation:* a development concept which defines the voluntary contribution people make in public development programmes through their involvement in various stages—decision-making, implementation and evaluation. It is an active process where those involved use economic and social resources to take action and make decisions to achieve their objectives. Thus, taking part in the development process from the bottom-up perspective (Nikkhah & Redzuan, 2009).

*Value chain:* the full range of activities and services needed to bring a product or service from its conception to end-users. Value chains also include the entire network of actors involved in input supply, production, processing, marketing, retailing, and consumption, operating within a complex environment that makes up the broader agri-food system (Christiaensen et al., 2021; Gereffi & Fernandez-Stark, 2011; Haggblade et al., 2012; Osti et al., 2015; Webber & Labaste, 2009).

*Youth:* persons aged between 15 and 35 (African Union, 2006).

### 1.2 Background on youth value-chain participation in South Africa and KwaZulu Natal

The South African (SA) government has introduced various initiatives to develop agri-businesses and promote value-chain participation by smallholder farmers and previously marginalised communities in the mainstream economy. Examples include the Agriculture and Agro-processing Master Plan (AAMP), Agro-Processing Support Scheme (APSS), Agri Hubs, and the Agro-processing Competitiveness Fund (APCF). These initiatives are funded through the National Empowerment Fund (NEF) and the Comprehensive Agricultural Support Programme (CASP). Implementing the AAMP links emerging rural producers of farm and non-farm products (especially women and youth) to markets and appropriate value chains. AAMP also facilitates skills development through targeted capacity building and enterprise development support while promoting procurement policies favouring women and youth. This includes capacitation in value-chain activities such as food and beverage packaging, fruit packaging and grading, meat processing, and food and beverage services (DALRRD, 2021). The AAMP also highlights plans for developing infrastructure in KZN for field crop value chains. This includes irrigation, roads, milling plants, storage and warehouse facilities for grain and oilseeds (DALRRD, 2022).

During the 2020/2021 financial year, the Department of Agriculture, Land Reform and Rural Development (DALRRD) provided skills development opportunities to 1 926 youths through the National Rural Youth Service Corps (NARYSEC) program. From this, 554 completed training in agricultural-related learning programmes spanning a range of commodities such as beef, dairy, maize, piggery, poultry, vegetables, and others. Moreover, since 2010, 4 071 youth have been trained in agricultural-related learning programmes through the NARYSEC Programme in various provinces across SA (DALRRD, 2021). Although progress is being made, significant challenges associated with value-chain participation by smallholder farmers and communities still exist. Such challenges include difficulty obtaining the necessary knowledge and skills, inadequate business management training and lack of empowerment (Khoza et al., 2019; Mdoda & Christian, 2021).

Additionally, most agro-processing and storage technologies require significant investment and scale, thus, favouring larger commercial farmers over smallholder farmers (Shirley, 2017). Nonetheless, SA is still experiencing challenges related to the collapse of infrastructure and services in small towns and general service delivery—including electricity, roads and water and the lack of well-functioning municipalities (BFAP, 2021), thus, limiting the effectiveness of investments made in agriculture and agro-processing. Furthermore, research highlighting youth’s negative perceptions about agriculture in SA associates these perceptions with the social stigma attached to the industry, portraying farming as a career for the poor and old with limited financial returns, high risks and uncertainty, and little success (Magagula & Tsvakirai, 2020; Metelerkamp et al., 2019; Swarts & Aliber, 2013). The factors shaping youth’s negative perceptions mainly focus on the primary sector and not other activities throughout the value chain. This highlights a lack of exposure to value chain activities and information on value chain careers. Zwane and Van Niekerk (2017) and Henning et al. (2022) recommend that policymakers develop supportive policies to attract and retain young people in agriculture while exposing them to initiatives focused on the value chain. The exposure should also include support programs and the opportunity to gain experience in the agricultural sector.

## 2 Methodology

The first section of the methodology (cf., section 2.1) reports on the study’s design, which was conducted in two phases. Phase 1(cf., section 2.2 to 2.1.4) reports on the methodology used to guide the focus group discussions. This includes an explanation of the study site in section 2.2, the data collection process in section 2.3 and focus group data analysis in section 2.4. The second phase of the data collection process is reported in Sections 2.5 (survey data collection) and 2.2.2 (survey data analysis).

### 2.1 Study design

The study was conducted in two phases. Focus Group Discussions (FGDs) were conducted in the first phase to understand youth awareness of value-chain opportunities and their perception of their role in the value chain. Upon analysing the results of the FGDs, questions were formulated for the second phase to gain insight into the dynamics of youth empowerment in agriculture and rural development from stakeholders’ perspectives based on the challenges and opportunities arising from the FGD data. An online survey was conducted with agriculture and rural development stakeholders in SA at a national and provincial level. The data collection was conducted following the requirements of the University of KwaZulu-Natal’s Human and Social Sciences Research Ethics Committee (protocol reference number: HSSREC/00002355/2021).

### 2.2 Phase 1: FGD study site

The FGDs were conducted in three communities in the KwaZulu-Natal (KZN) province, SA, between June and September 2021. The KZN province is dominated by the black African population (87,0%), where IsiZulu is the language most spoken at home, followed by English (Stats, 2018). KwaZulu-Natal is the province with the second largest population in SA after Gauteng, with an estimated 11.5 million people (Stats, 2021b). Approximately half the population is concentrated in the working-age (aged 15–59 years) bracket for all municipal districts in KZN (Stats, 2018). Additionally, the KZN province is the second largest contributor to agricultural employment in SA, employing approximately 105 076 (13.1%) employees. KwaZulu-Natal’s mild sub-tropical climate provides comparative agriculture and tourism advantages (Stats, 2021a). The selected sites were Swayimane, Nhlazuka, and uMbumbulu communities. These communities reflect various socio-economic contexts where youth in SA are located. They have different social and environmental indicators and varying exposure to the concentration of poverty, unemployment, and economic prospects—the selected sites as described further in Table 1. The sample size for the youth FGDs was 72, allowing three FGDs to be conducted in each community, with each FGD ranging from six to eight participants. According to (Guest et al., 2017), over 80% of all themes are discoverable within two to three focus groups, and 90% are discoverable within three to six focus groups. Thus, conducting three FGDs per community was based on the minimum number of FGDs sufficient to identify the most prevalent themes within the data set and resource availability.

### 2.3 Phase 1: FGD data collection

Seven FGDs were conducted, with each group ranging from six to eight participants. FGDs usually consist of a small group of people between six and ten who meet to express their views about a topic defined by a researcher (Cronin, 2008). These discussions aimed to understand the youths’ awareness of value-chain opportunities and their perception of their role in the value chain. Therefore, based on the purpose, FGDs were most useful for generating information on collective views and understanding participants’ experiences and beliefs (Gill et al., 2008). During data collection, the FGDs were only conducted with the youth and the online survey questionnaire was only administered to stakeholders. Three FGDs were conducted in each of the uMbumbulu and Nhlazuka communities, and thematic saturation was reached; therefore, only one FGD was conducted in Swayimane to include the views of youth from a different community. Out of the seven FGDs conducted, five groups represented males and females well. The FGD participants were recruited using a purposive sampling method, where selection is according to the study objectives (Cronin, 2008). The participants who participated in the FGD were 18 to 35 years old. The category of the FGD participants was a combination of youth involved in agricultural groups in their communities, youth interested in farming, and youth who assisted their parents with farming activities. Before data collection began, a gatekeeper permission letter was obtained from the traditional councils in each community. During data collection, the FGD facilitators obtained informed consent from all participants to record the FGDs and take images of the mapping exercise for data analysis. All activities were conducted in isiZulu, the first language of the participants.

The FGD were conducted and transcribed by two facilitators recruited as research assistants for data collection. The researcher was present during data collection as an assistant and observer. The FGD began with an exercise to map the agriculture value chain based on the youths’ understanding. This mapping exercise investigated youth awareness of value-chain employment opportunities and their perception of their role in the food system. Each group of participants was given images representing the different parts of the agriculture value chain (inputs, production, harvesting, food processing, packaging, marketing and retail) and 10 minutes to develop value chain maps. The discussion continued, and participants were asked about their knowledge of the agricultural value chain and the difference between agricultural and farming careers. Then, a second set of the same images of agricultural value-chain activities was given to the participants. The FGD facilitator divided the flip chart paper into two parts. The first part was labelled “most employment opportunities”, and the second was labelled “least employment opportunities”. The participants were then asked to stick the images under the appropriate heading while explaining their choices. The discussion concluded with the facilitator requesting the participants to provide their perspectives on agricultural programs targeting young people, their views on who should be responsible for attracting youth to agriculture, and what those people/stakeholders identified as responsible should do to promote interest in agriculture amongst youth.

### 2.4 Phase 1: FGD data analysis

The study adopted the framework analysis approach that Spencer et al. (2003) developed for analysing qualitative data. This approach was initially developed in the late 1980s for large-scale policy research. It is suitable for research with specific questions, a limited time frame and a pre-designed sample (Srivastava & Thomson, 2009). It can also be used with various narrative data collection methods such as interviews, focus groups, observation and documentary analysis (Ritchie et al., 2003). This analysis approach involves distinct but interconnected stages: familiarisation, identifying a thematic framework, indexing, charting, mapping, and interpretation. Although this analysis approach is thematic, it has flexibility and allows for developing themes from the research questions and the narratives of research participants (Gale et al., 2013). Therefore, findings of this type of analysis are mainly represented based on emerging key themes and not using descriptive statistics. The following procedure was followed during data analysis.

#### 2.4.1 Stage 1: Familiarisation

The researcher (first author) listened to the FGD recordings whilst reading the transcripts and observational notes that the facilitators prepared. The researcher aimed to be familiar with the data, get a sense of the whole discussion, become aware of critical ideas and recurrent themes, and note them before breaking the data into parts (Ritchie et al., 2003; Srivastava & Thomson, 2009). The researcher uploaded the transcripts into the QSR NVivo program, which helps sort, manage and analyse qualitative data (QSR International, 2015). Moreover, Parkinson et al. (2016) found that conducting the framework analysis in NVivo improves the transparency of the analysis process because it leaves a clear audit trail, so analytic decisions and interpretations can be easily traced back to the raw data.

#### 2.4.2 Stage 2: Constructing a theoretical framework

This stage of framework analysis aimed to organise data in a meaningful and manageable way for subsequent retrieval, exploration, and examination during the final mapping and interpretation stage (Parkinson et al., 2016). The emerging themes and concerns noted from the earlier stage also informed this process. The researcher again carefully read the transcripts line-by-line, taking note of codes and themes arising in the data using the “nodes” function in NVivo. Developing themes is a common feature of qualitative data analysis, involving the systematic search for patterns to generate full descriptions capable of shedding light on the phenomenon under investigation (Gale et al., 2013).

The main themes, however, had been predetermined from the research questions. These themes were: attracting youth into agriculture; employment opportunities in the agriculture value-chain, programs for youth in agriculture; understanding the value-chain activities; and farming and agriculture careers. The additional themes developed from participants’ narratives were: recommended actions for attracting youth into agriculture; and participants’ experiences with agricultural programs targeted at youth. These developing themes were also coded under “nodes” in NVivo. After coding a few transcripts, an operational, analytical framework that included all the codes was developed and applied to all subsequent transcripts. After that, a revised base framework of categories emerged from the key areas of interest in the study based on the objectives (see supplementary information Table S1).

#### 2.4.3 Stage 3: Indexing, charting, and mapping

Data from the transcripts were organised into the framework categories and then under the “cases” function in NVivo. In NVivo, the term “case” is used as a unit of analysis within a study that can be further used as a foundation for building theories or making claims (QSR International, 2015). Cases which represent various topics emerged during the analysis. For example, exposure (rural versus urban areas); minimum work requirements; social connections and networks; and youths’ social status. These cases were associated with the “most employment opportunities” code. At the end of this stage, the indexed data was organised into a more manageable format to facilitate the analysis in the next step.

#### 2.4.4 Stage 4: Interpreting the data

Data were further analysed under the different codes/themes during this process, including comparing the cases and sorting out quotes representing the main categories. Furthermore, the main themes emerging from the data were categorised based on research objectives and are reported in the results section. Lastly, mapping connections between the themes to explore relationships and causality further was also done in NVivo.

### 2.5 Phase 2: Survey data collection

The survey was conducted online using Google Forms, a convenient and efficient online platform to administer questionnaires to respondents around the clock and anywhere using a unique URL link (Vasantha Raju & Harinarayana, 2016). Based on the number and geographical distribution of the target population (*n* = 146), an online survey was ideal to limit personal contact and adhere to the COVID-19 regulations prevailing in SA at that time. This survey formed part of research conducted in partnership with the Food and Agriculture Organization of the United Nations (FAO), the National Department of Agriculture, Land Reform and Rural Development (DALRRD), and the University of KwaZulu-Natal (UKZN). A list of stakeholders was requested from DALRRD. This list was inclusive of 146 members representative of various provincial departments of agriculture, youth programmes’ coordinators (programmes directed at youth empowerment; youth in agriculture; and rural development), international structures/agencies, national organisations/structures, state-owned entities, industry bodies, financial sector, provincial youth in agriculture committee representatives, and academia. Based on their mandate and line of work, these stakeholders hold valuable insights and inputs into the dynamics of youth empowerment in agriculture and rural development in SA.

The survey questions were formulated partly based on the emerging themes from the FGD’s data. The survey included single, multiple-choice, open-ended, and Likert scale questions. The survey consisted of five brief sections and took approximately 10–15 minutes to complete. Stakeholders were invited to an email group on Microsoft Outlook, where an invitation to participate in the survey was shared. The survey was open for two months, and a survey reminder email was sent to stakeholders biweekly. However, the survey response rate was only 29,5% (*n* = 43), which could be attributed to survey fatigue. Since the beginning of COVID-19, there has been a rise in the number of online surveys conducted within a short period, and respondents have become reluctant to complete surveys altogether (De Koning et al., 2021; Hathaway et al., 2021). According to Nulty (2008), online surveys are much less likely to achieve response rates as high as surveys administered on paper—on average, 33% compared with 56% on paper-based surveys. Moreover, other researchers (Conrad et al., 2010; Sax et al., 2003; Van Mol, 2017) have also experienced low response rates of 30% and less on online surveys. However, given the survey design, its clear focus, and that surveyed individuals provided institutional perspectives, we believe that 43 respondents provided a sufficient sample size for analysis and interpretation.

### 2.6 Phase two: Data analysis

The survey results were analysed using a combination of descriptive, quantitative and qualitative analyses:

(a) Google Forms: generated basic analyses in percentages, pie charts, and bar graphs.
(b) Quantitative analysis: the IBM Statistical Package for Social Sciences (SPSS) Version 27 (IBM Corp, 2020) was used for generating descriptive statistics, frequencies, and analysing relationships using crosstabs analysis and a Chi-square of the data generated from multiple-choice questions.
(c) Qualitative analysis: Open-ended questions were analysed using QRS NVivo (QSR International, 2015) to identify emerging themes and patterns between themes.

## 3 Results

The results are organized based on the objectives of this study. The first part (Sections 3.1 to 3.1.3) reports the results of the youth’s awareness of value-chain opportunities and their perception of their role in the value chain. Sections 3.4 to 3.1.6 characterise the dynamics of youth empowerment in agriculture and rural development from a stakeholder’s perspective.

### 3.1 Employment opportunities in the agricultural value-chain

**Figure 1:**
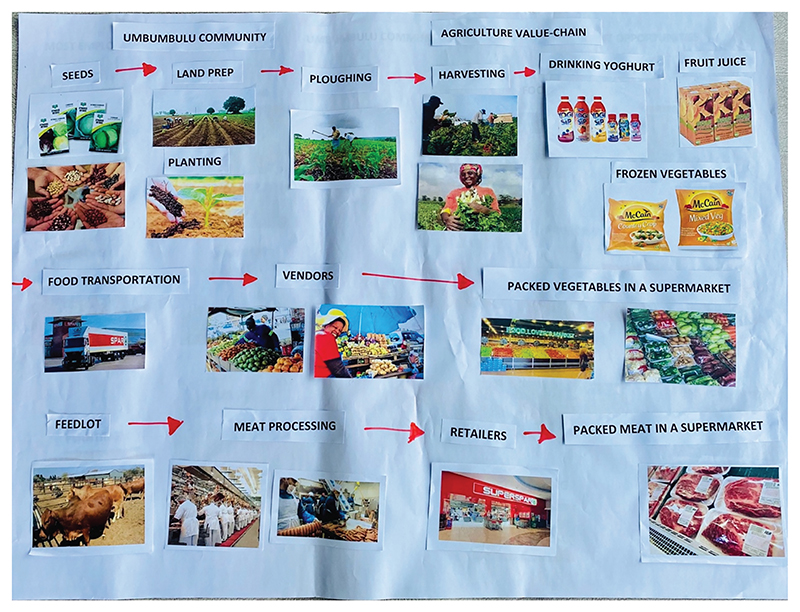
An example map from the first mapping exercise of value chain activities, developed by focus group participants from the UMbumbulu community (value chain activities listed from left to right in sequence).

The youth had some knowledge of value chain activities and the sequence of these activities. During the mapping exercise, four groups opted to map the crop agricultural production value chain compared to animal production, but three groups mapped both (Figure 1).

Across all communities, most participants were not knowledgeable about other activities and careers besides primary agricultural production. This could be attributed to participants only being exposed to value-chain activities in their local surroundings. For example, after the mapping exercise, some responses were as follows when asked about the difference between agricultural and farming careers.

”.It’s difficult to think of careers related to things you do not understand or aren’t involved in. I only started thinking of other agriculture careers when we did this exercise. We understand agriculture based on primary production activities because it’s what’s happening around us.”-– Source: Field Survey, 2021(uMbumbulu, FGD 3)

”…The availability of employment opportunities in rural areas versus urban areas is different. For example, for us in rural communities, there are employment opportunities in farming, but someone in the city, for example, in Durban, is more exposed to employment opportunities in the processing sector and not primary agriculture.” – Source: Field Survey, 2021(Nhlazuka, FGD 1)

**Figure 2:**
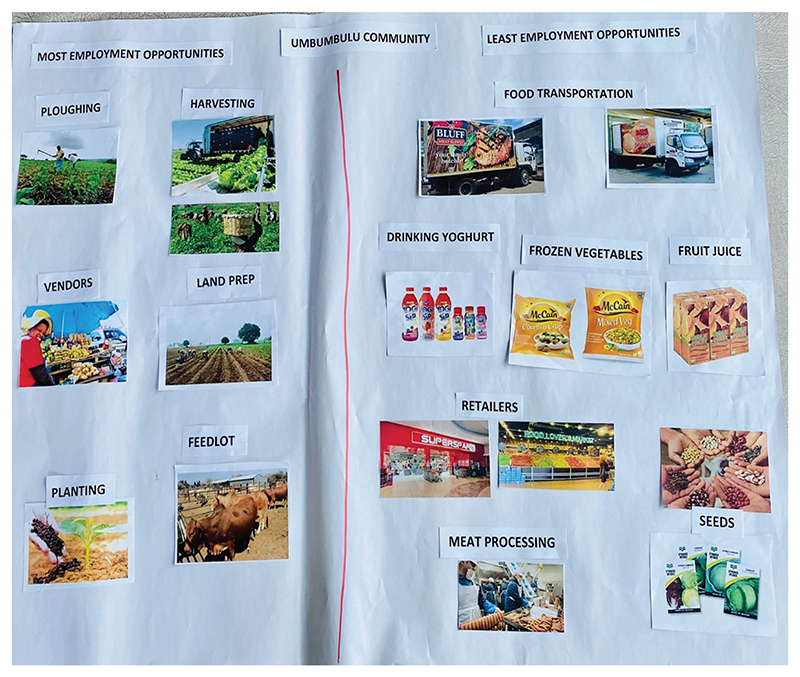
An example map of the second mapping exercise of employment opportunities in the agricultural value chain, as identified by focus group participants from the uMbumbulu community.

**Figure 3:**
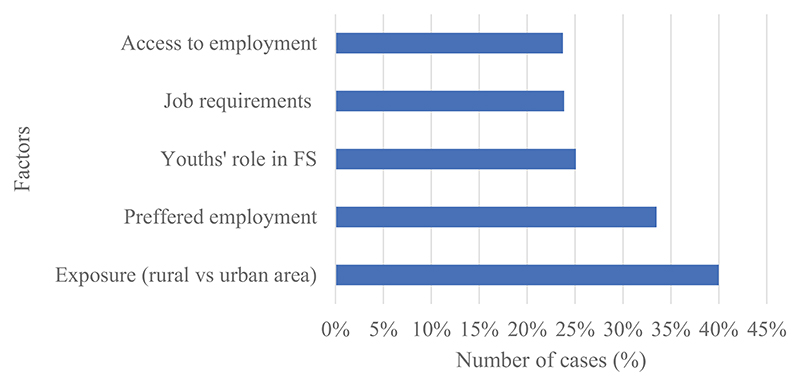
A graphical representation of factors linked to youth’s exposure to agricultural value chain activities based on their current involvement in agriculture. This graph was generated in NVivo, and the factors were based on topics emerging from the coded data (nodes).

Correspondingly, the participants categorised the primary sector as having the most available employment opportunities compared to other sectors in the value chain (Figure 2). In addition to exposure in rural areas versus urban areas, other factors linked to youths’ perceptions of employment opportunities in the value-chain (see Figure 3) were their preferred type of employment in agriculture, the current roles youth occupy in the value-chain, job requirements for available opportunities, and their overall access to work. The participants who participated in the FGDs were a combination of youth involved in agricultural groups in their communities, youth interested in farming, and youth who assisted their parents with farming activities.

Most youths showed interest in agricultural activities that are less labour-intensive and with a shorter return on investment period (see Figure 3). The labour intensity of the farming activities that some respondents were involved in was a key challenge. The respondents pointed to minor injuries, severe blisters, long hours spent in the field, and low rewards for their hard work. This was also linked to the limited exposure to value-chain activities in their surroundings. The participants were based in peri-urban and rural communities, where many agricultural operations were still labour-intensive with limited returns. Some of the responses that reflected this are as follows.

… hoeing and de-weeding are hard. I don’t have gloves, which has damaged my hands. So I would like to do something that is not as labour intensive and something more ideal for young people. I’m interested in processing and marketing. - Source: Field Survey, 2021 (uMbumbulu, FGD 3)

We grew up around farming, and it is annoying. But we would like to be trained and educated in agriculture so we can have something to show for it, you know. We cannot keep waking up early in the morning to go to the fields and planting repeatedly, and that’s where it ends. It’s annoying and tiring. We would like the government to at least consider adding training and ensuring that we can get certificates or accreditation in something related to agriculture so we can be employed somewhere, not just being here planting crops. - Source: Field Survey, 2021 (Nhlazuka, FGD 3)

Additionally, most of the youth in all FGDs highlighted that they are only exposed to employment opportunities in primary agriculture. For one to be employed on a farm, the job requirements are simple, and no qualifications or resumes are required. Whereas, when one seeks employment in other parts of the value chain, such as processing and retail, the job requirements are different, and most youths do not qualify to apply because of a lack of requisite skills. Hence, the request for training.

### 3.2 Agricultural programs targeting youth

Most of the respondents had negative perceptions about agricultural programs targeting youth. Respondents described these programs as having “empty promises; slow; not doing enough of what they require; and not being progressive”. Moreover, one’s social network increases the chances of participating in an agricultural program and accessing resources and employment opportunities. For example, some responses were as follows,

The department does not give us anything. No assistance, no useful information or idea of what you do. We’ve seen the equipment, implements, and things the government has donated, but no one benefits from them. You go there, ask for assistance, and they take your farm details, but nothing happens. There have been tractors that have parked in this community since I was in primary school; who have those tractors helped? - Source: Field Survey, 2021 (uMbumbulu, FGD 3)

“The issue is they won’t agree for us to farm and do our own thing. They always want us to partner up and form groups. So that’s hard, it’s better to work alone. You see progress.” - Source: Field Survey, 2021 (Nhlazuka, FGD 3)

**Figure 4:**
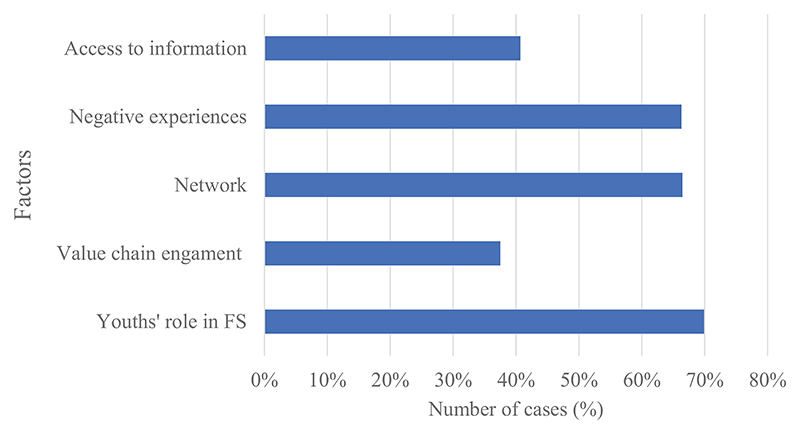
A graphical representation of additional factors related to challenges associated with local agriculture programs targeted at youth. This graph was generated in NVivo, and the factors were based on topics emerging from the coded data (nodes).

Other challenges associated with agricultural programs targeting youth (see Figure 4) were lack of access to information, the programs target engaging youth in aspects of agriculture they are not interested in, and the programs not related to youths’ preferred jobs within agriculture.

### 3.3 Attracting youth into agriculture

Across all the focus groups, the respondents’ recommendations for attracting youth into agriculture were linked to their experiences and interests. The leading suggestions were improving: (i) access to information; (ii) increasing youth exposure to other value-chain activities and employment opportunities; (iii) provision of training programs that will enhance their chances of success in the industry; (iv) provision of training and information on aspects of agriculture youth are interested in; and (v) giving young farmers a voice in policy formulation and implementation. The respondents also highlighted using social media advertising to attract youth into activities.

**Figure 5:**
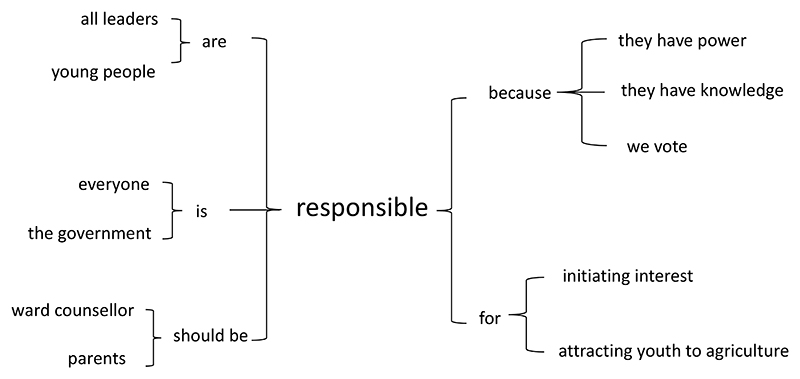
Word tree showing the key phrases associated with ‘responsible’ in the coded data set of all FGD. This word tree is related to who is responsible for attracting youth into agriculture. It was used as the foundation in exploring who the respondents believe is responsible for attracting youth into agriculture.

Further analysis showed that the respondents felt everyone is responsible for attracting youth to agriculture. This includes government leaders, ward counsellors, young people, parents, and community members (see Figure 5). The respondents felt that government leaders are responsible as they have been elected. Young people who have succeeded in the industry are also responsible for guiding and attracting peers. As all the respondents came from communities where agriculture is dominant, respondents felt that parents and older women are more knowledgeable about farming and should provide support and guidance.

### 3.4 Stakeholder institutions

This section provides an overview of the respondent’s profiles from the online surveys. It also briefly highlights the type of institutions the respondents worked for, the kind of youth initiatives emanating from those institutions, the support institutions provide with youth initiatives, the mode of advertisement, and the eligibility criteria for participation (Table 2).

A combination of employees from different sectors participated in the survey (Table 2). Most (55.8%) of the stakeholders had been employed in their current organisations for more than five years, with only 27.9% who had been with their current organisations for less than a year.

Some (53.5%) of the organisations where the respondents worked run youth programs/initiatives. These initiatives are mainly focused on: (i) skills development and training; (ii) small enterprise development; (iii) promoting entrepreneurship; and (iv) employment placement in the agriculture industry. The eligibility criteria of most programs/initiatives were: SA citizenship; residence within the program/initiative implementation area; willingness to learn; interest in agriculture; and the relevant university qualification for employment placement programs and involvement in farming for entrepreneurship programs. These initiatives were mainly advertised through social media platforms, organisation websites and networks, agricultural-related gatherings, meetings, and word of mouth. Only programs and initiatives implemented by the government are advertised on print media and radio. The majority (43.5%) of the programs/initiatives had a duration of 12 months or less, with 39.1% lasting between 2–3 years and 17.4% going on for longer than five years. Moreover, most (69.9%) of these programs were mainly suited for entry-level careers, with only 34.8% for all career levels.

### 3.5 Youth employment and empowerment in agriculture and rural development from a stakeholder perspective

**Figure 6:**
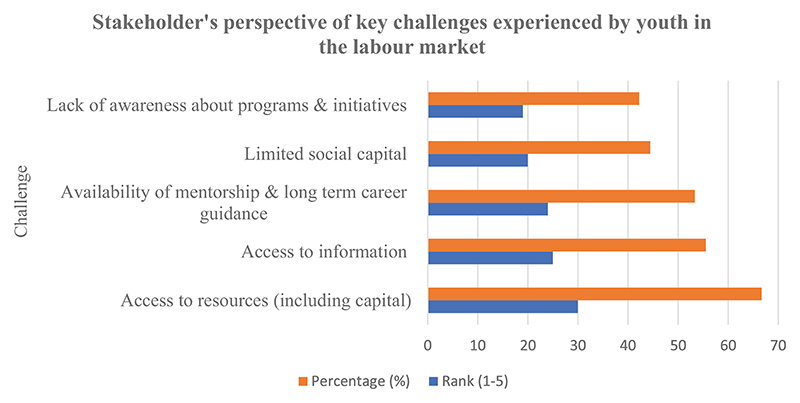
A graphical representation of stakeholders’ perspectives of youth’s challenges in the labour market. Stakeholders were asked to rank challenges on a scale of 1 to 5 (least to most). This graph was generated based on data collected using Google Forms

Most (74.4%) stakeholders believe that youth support services and development programs were mainly in the primary sector (see Figure 6). Most initiatives and support from the government, donors and non-governmental organisations were geared toward primary agriculture. For example, supporting farmers with inputs, equipment, and extension services promotes higher yields. Youth also had access to advice and support from parents and community members when starting businesses. However, these community members usually only have experience in primary agriculture. Moreover, stakeholders indicated that, compared to other sectors, primary agriculture had low entry requirements, requires less capital, and has accessible training initiatives for various levels and scales of farming.

Although the secondary sector (37.2%) is perceived to have the least support services, the rest of the stakeholders were nearly equally distributed, indicating that the primary (32.6%) and tertiary sectors (30.2%) also have limited support services (Figure 6). The distribution of stakeholders’ opinions on the availability of support and development programs for youth in the value chain indicates a deficit of support services in the secondary (20.9%) and tertiary sectors (4.7%) (Figure 6). Moreover, the secondary sector also requires specialised skills and knowledge, creating a barrier to entry, difficulty accessing markets, and a lack of available information on value-adding and processing operations; the sector is poorly developed and funded.

### 3.6 The relationship between youth participation in agriculture, perceptions, aspirations and access to resources and information

A crosstab analysis explored possible associations between stakeholders’ opinions about factors linked to youth participation in agriculture against the stakeholder’s job title and employment period (dependent variables). The independent variables were the youth’s perception of the industry, aspirations and interests, access to resources, information, social network, and locality, see example in Table 3. No statistically significant relationships were found between the dependent and independent variables (Chi-Square: 14.65, *n* = 43, df = 16, *p* = 0.551). However, based on the distribution of responses, a majority (81.4%) of the stakeholders, irrespective of their job title and years of employment, agree that youth participation in agriculture is associated with youth access to resources (81.4%) or information (86%).

The X^2^ test is not statistically significant. Additionally, most stakeholders, regardless of job titles, agreed that social networks influence youth awareness of various agricultural programmes (74%) (refer to Table 4, Chi-square: 8.441, *n* = 43, df = 16, *p* = 0,935).

**Figure 7:**
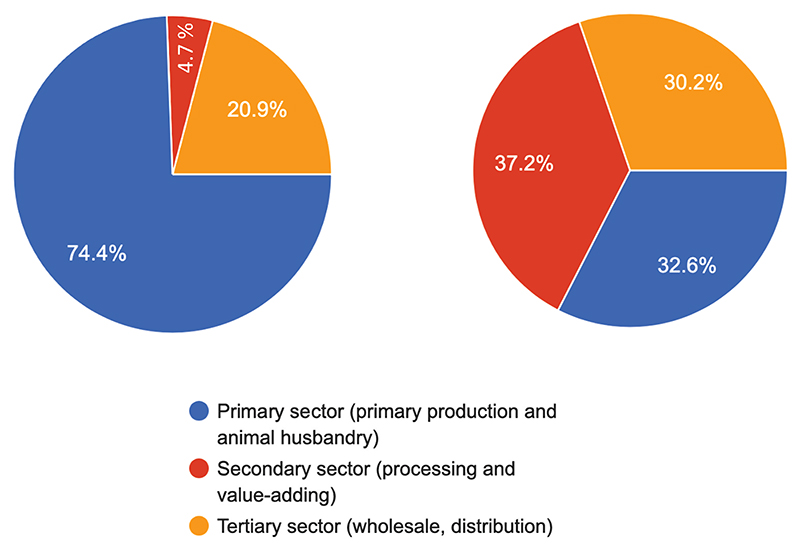
Distribution of stake-holder’s opinion of which sector of the agricultural value-chain has the most support services (panel A, left hand side) and the least support services (panel B, right hand side).

Based on the stakeholder’s perspectives (see Figure 7), the five critical challenges experienced by youth in the labour market from stakeholder’s perspectives were ranked as (i) access to resources and capital (69.8%); (ii) access to information (58.1%); (iii) availability of mentorship and long-term career guidance (55.8%); (iv) limited social capital (46.5%); and (v) lack of awareness about programs and initiatives (44.2%).

## 4 Discussion

The study investigated youth awareness of value-chain opportunities, perceptions of their role in the value chain and their perception of agricultural programs targeting youth. In addition, it sought insight into the dynamics of youth empowerment in agriculture and rural development from stakeholders’ perspectives.

The results suggest that youth lack awareness of value chain careers and are only exposed to primary agriculture in their communities. Additionally, there is a deficit in information, skills training, mentorship, long-term career guidance or support for youth regarding value-adding activities. Similarly, Mulema et al. (2021) also found a lack of agriculture-related skills, market access, and functioning infrastructure as key market factors pushing the youth away from participating in agribusiness in Vietnam and Zambia. Value-chain participation is essential for developing rural enterprises and the rural economy. Moreover, agro-processing activities can address socioeconomic challenges, provide additional sources of income, create employment opportunities, contribute to food security and enhance livelihoods (Olaoye, 2014; Woodhill et al., 2022).

Importantly, supporting youth to transition into new livelihood opportunities requires improvements in access to information, social networks, connectivity to markets, skills and training, access to productive resources, finance, political participation, and empowerment (Giuliani et al., 2017; Khoza et al., 2019; Moore, 2015; Ng’atigwa et al., 2020; Sumberg et al., 2012). Furthermore, investing in developing rural infrastructure (roads, reliable electricity, storage facilities, and digital networks) and providing basic services to ensure communities are not isolated can promote value chain development (International Fund for Agricultural Development, 2021; Khoza et al., 2019). Without addressing the critical barriers to entry, young people will remain excluded and unable to take advantage of value chain opportunities and diversify their livelihoods.

Most youths showed interest in agro-processing and agricultural activities that are less labour-intensive and with a shorter return on investment period. However, the available support is short-term, suitable for entry-level careers, and concentrated in the primary sector. Additionally, it is mainly advertised online, at agricultural-related gatherings or meetings and by word of mouth. Youth in remote rural areas or those with weak social networks are excluded. Ng’atigwa et al. (2020) found that public-private partnerships could increase youth awareness, knowledge, and access to innovations, through formal education and agricultural extension services, consequently encouraging youth participation in agribusiness. Off-farm activities in food value chains and support services have the potential to offer aspirational and diverse employment opportunities for youth in input supply, digital technologies, processing, packaging, storage, distribution, or retail (Knobloch & Pirzer, 2020; Owoo & Lambon-Quayefio, 2017). However, for youth to engage in food system activities, opportunities and relevant support need to be accessible (International Labour Organization, 2019). The government is key in driving and influencing change and transformation through policy development. However, at the local level, policy interventions are implemented by a broad range of actors (International Fund for Agricultural Development, 2021).

Furthermore, diverse youth experience varying levels of food system employment opportunities based on their access to financial, physical, social, human, and natural capital (Bullock & Crane, 2021; Fox & Kaul, 2018; Knobloch & Pirzer, 2020). Also, their diversity is associated with additional factors such as age, gender, ethnicity, and educational background. This diversity of youth context influences their engagement in agriculture and their capacity to take advantage of food system opportunities (Chipfupa & Tagwi, 2021; LaRue et al., 2021). Consequently, considering such heterogeneity requires an inclusive approach to transforming the food system and unlocking opportunities for youth. Importantly, as the International Fund for Agricultural Development (2021) highlighted, good governance and effective policymaking are informed by up-to-date comprehensive evidence. Despite growing knowledge of youth affairs across sub-Saharan Africa, numerous governments still lack extensive evidence or valuable insights on youth heterogeneity concerning agri-food systems aspirations, opportunities and challenges (LaRue et al., 2021; Sumberg et al., 2012). Consequently, it is challenging to develop and implement responsive or effective policies.

## 5 Limitations

The results of the study should be considered in light of some limitations. Although the sample size was significant for statistical analyses (*n* = 43, response rate 29,5%) and nationally representative, it may not be a large enough sample to allow for broad generalizations. Moreover, future research should widen the target sample by including a more comprehensive range of youth involved in agriculture in South Africa. Additional questions related to youth participation in agriculture could be included in the routine national surveys Stats SA conducts. This would ensure a more reliable and frequently updated dataset to track and monitor youth participation in agriculture.

## 6 Conclusion

The study sought to investigate youth awareness of value-chain opportunities and their perception of their role in the value chain. The study also sought insight into the dynamics of youth empowerment in agriculture and rural development from stakeholders’ perspectives.

Youth lack awareness of value chain careers and opportunities. Although most youths in the study were interested in agro-processing and value-chain activities, available support is mainly dedicated to the primary sector and suited for entry-level careers. Moreover, youth also lack the necessary skills and training to work in the secondary sector. Usually, the constraints limiting value chain benefits in addressing socioeconomic challenges result from barriers to entry for the beneficiaries. Addressing the key barriers to entry, which in this case is capacity building through improving access to information, increasing youth’s exposure to value-chain activities, and providing skills training will enhance chances of success in the industry.

Additional challenges limiting youth participation in value chain activities include a lack of access to information, weak social capital, and an underdeveloped and poorly funded agro-processing sector. Diversifying from primary production into other activities in the value chain and support services is imperative to unlock decent employment opportunities for youth. Value-chain promotion is usually one component of a more extensive programme that contains various interventions. Therefore, priority must be given to all value chain sectors to avoid skewed investments. Moreover, promoting awareness to improve youth access to information and exposure to value-chain careers is essential. Government partnerships with value-chain actors, civil society, academia, and public-private institutions in the agri-food system can promote value-chain activities, attract youth into the industry, and strengthen available support systems for broader impact.

Youth involvement in the agriculture value chain has the potential to unlock jobs and contribute to addressing socio-economic challenges throughout Africa. The main challenges preventing youth from participating in agriculture in various parts of Africa are similar. Although this study is South African-centric, the study’s findings can be used to shed light on the importance of youths’ perception of their involvement in value chain activities. Moreover, to examine the investments made in agriculture awareness and where current resources are directed in value chain activities.

## 7 Recommendations

Based on the evidence presented in this study, the following are recommended:

- More effort should be invested in agribusiness awareness programs to increase youth exposure to value-chain activities, access to information and career awareness. Policymakers should integrate agriculture and food systems knowledge into the primary education curriculum to promote youth awareness and evoke interest in agri-food system careers at an early age. Public and private agriculture extension support and advisory services should also reorganise themselves to offer such information and expose young people to opportunities across the value chain.
- Investing in ICTs and telecommunication infrastructure development in peri-urban and rural areas can improve access to information, knowledge exchange and networking amongst youth and value-chain actors. For example, radio stations can be an awareness and knowledge-sharing platform for youth in remote rural areas. This could assist in value-chain awareness, sharing opportunities and innovations, and youth engaging more effectively in value-chain and food systems dialogues.
- The government should leverage public-private partnerships to increase access to long-term business support for agribusiness youth, especially in remote rural areas.
- National statistical data on youth engagement in agriculture and food systems need strengthening. A reliable and regularly updated database would aid policy making and guide the direction of investments. Additionally, further research must be conducted to map the effectiveness, scope and impact of agricultural programs targeting youths. Understanding the dynamics of youth empowerment and participation in agribusiness will also assist policymakers in formulating relevant and responsive policies.
- Government programming needs to prioritise all value-chain activities and not only primary agriculture. Investments should be distributed evenly to create employment opportunities for youth throughout the value chain.
- Agriculture transformation policies need to be more holistic and adopt a food system thinking perspective to promote youth participation in value chains. Farming systems consist of various commodities and livestock with different value chains. Therefore, agricultural transformation policies must leverage existing synergies and interdependencies of various value chains when designing interventions to promote systemic transformation. Moreover, adopting a system-thinking approach will promote evenly distributed investments in the agri-food system and unlock diverse employment opportunities for youth. However, further research must be conducted to compare the impact of holistic and targeted agriculture transformation policies on employment creation.
- Unlocking opportunities for youth in value chain activities calls for coordination and collaborations among various government sectors, the private sector, academia, civil society, and youth organizations in shared planning and implementation of interventions. Moreover, the provision of incentives by the government and investment in agribusiness incubators could enhance youth employment by assisting them in preparing for agribusiness and value chain participation (Etela & Onoja, 2017; International Labour Organization, 2019). Agribusiness incubators can play a role in assisting entry-level enterprises and entrepreneurs with business development support, access to information, agricultural technology, networking, capital, marketing, logistic support, training, mentorship, infrastructure and other facilities (Rukarwa et al., 2018; Segawa, 2019; Singh, 2014).

## Supplementary Material

Supplementary materials

## Figures and Tables

**Table 1:** Background information of sites and brief description based on information from the provincial profile statistics (Stats, 2018)

Site	Socio-economic context	Total population at the local municipality level	Youth population (ages 15 to 34) at the local district municipality level	Main economic sectors*
Swayimane, Ward 8 (approximately 65km from the capital city, Pietermaritzburg, in uMshwathi Local Municipality) GPS coordinates −29.490003120299047, 30.65599300942323	Located in underdeveloped areas with limited access to basic physical and social requirements, very few economic opportunities, poverty and inequality (UMshwathi Municipality, 2020)	111 645	38 902	Agriculture (subsistence farming, small-scale and scattered pilot projects of cooperative sugarcane farming). Commercial farmlands are predominately white-owned sugarcane and timber (UMshwathi Municipality, 2020).
uMbumbulu, oGagwini area Ward 96 (a peri-urban zone located approximately 45 km Southwest of Durban in the South-Eastern part of KZN) GPS coordinates −29.982566340076584, 30.70292641113637	It is the largest Tribal area within eThekwini Metropolitan Municipality. Characterised by poor service delivery, high levels of poverty and disease, and low levels of sustainable income or economic activities (Gilmore & Chasomeris, 2015)	3 702 231 (population at Metropolitan level)	1 568 378 (population at Metropolitan level)	Finance, manufacturing, and community services are the most significant sectors.^1^ There is some primary production from subsistence smallholder farming. However, it has declined with densification in the peri-urban landscape.
Nhlazuka, Ward 5 (a rural community in a tribal area, approximately 50km from Pietermaritzburg in the Southern part of the uMgungundlovu district municipality under Richmond local municipality) GPS coordinates −30.169629490352527, 29.96160876798539	Tribal authority areas characterised by a low level of basic services and facilities with substantial unemployment (Richmond Municipality, 2015)	71 322	24 313	Agriculture (subsistence farming, small-scale). Commercial farmlands are predominately white-owned sugarcane and timber, commercial agriculture, and subsistence agriculture, with a predominance of sugarcane and forestry.

**Table 2:** A summary of the respondent’s and institution profiles from the online surveys

Category	Frequency (*n*=43)	Percentage (%)
Type of organisation		
Academic institution	8	18.6
Community-based organisation	1	2.3
International intergovernmental organisation	1	2.3
Non-Governmental Organizations (NGOs)	10	23.2
Non-profit company	1	2.3
Private sector	4	9.3
Public sector	10	23.3
Public institutions	8	18.6
Job title		
Academia and research	10	23.2
Administrators, interns, and assistants	8	18.7
Advisors, facilitators, and fieldworkers	6	14
Executive management	13	30.2
Management	6	14
Years of employment		
(1) 0 -1 year	12	27.9
2–3 years	4	9.3
(1) 3 -4 years	3	7.0
Above five years	24	55.8
Institutions with youth initiatives		
Yes	20	46.5
No	23	53.5
Career level suitability		
Entry-level programs	16	69.6
Mid-career level programs	4	17.4
Advanced career level	1	4.3
All career levels	8	34.8
Program accessibility		
Rural youth	13	56.6
Peri-urban youth	10	43.5
Urban youth	2	8.7
Youth from all backgrounds	14	60.9

**Table 3:** Relationship between job title and access to resources

Job title		Access to resources					
		Strongly agree	Agree	Neutral	Disagree	Strongly disagree	Total
Academia and research	n	2	5	1	2	0	10
	%	11.1	29.4	25.0	66.7	0.0	23.3
Admin, interns and assistants	n	4	3	1	0	0	8
	%	22.2	17.6	25.0	0.0	0.0	18.6
Advisors, facilitators and fieldworkers	n	3	2	1	0	0	6
	%	16.7	11.8	25.0	0.0	0.0	14.0
Executive management	n	7	5	1	0	0	13
	%	38.9	29.4	25.0	0.0	0.0	30.2
Management	n	2	2	0	1	1	6
	%	11.1	11.8	0.0	33.3	100	14.0
**Total**	n	18	17	4	3	1	43
	%	100	100	100	100	100	100

**Table 4:** Relationship between job titles and stakeholder’s opinion on the influence of social networks on youth awareness of programmes

		Awareness of programmes					
Job title		Strongly agree	Agree	Neutral	Disagree	Strongly disagree	Total
Academia and research	n	3	4	2	0	1	10
	%	7	9	5	0	2	23
Admin, interns and assistants	n	5	1	1	1	0	8
	%	12	2	2	2	0	19
Advisors, facilitators and fieldworkers	n	2	2	1	1	0	6
	%	5	5	2	2	0	14
Executive management	n	5	4	2	1	1	13
	%	16	22	11	0	5	54
Management	n	4	2	0	0	0	6
	%	9	5	0	0	0	14
Total	n	19	13	6	3	2	43
	%	44	30	14	7	5	100
