# Supplementary material for: ‘Is there value for us in agriculture?’ A case study of youth participation in agricultural value chains in KwaZulu-Natal, South Africa
Source: Cogent Food Agric. Author manuscript; Available in PMC 2024 Jul 12. (PMC7616198; doi:10.1080/23311932.2023.2280365)
Supplement: Supplementary materials [file EMS197305-supplement-Supplementary_materials.docx]

**Supplementary information**

**Manuscript title:** ‘What’s the value for us in agriculture?’ A case study of youth and value chain participation in KwaZulu-Natal, South Africa

Table 1: Codebook/base framework used to code the focus group transcripts extracted from Nvivo

| **Code name/ nodes** | **Description** |
| --- | --- |
| Attracting youth into Agric | Participant's views about attracting youth into agriculture, who is responsible for attracting youth into the industry and views related to youth's role in agriculture |
| Employment opportunities in AVC | Participant's views on overall employment opportunities in the agricultural value chain |
| Experiences with Agric programmes | Participant's experiences with agricultural programmes |
| Farming and Agric careers | Participant's knowledge and understanding about the difference between farming careers and agriculture careers |
| Key observation employment in AVC | Key observations based on experience or opinions of participants related to employment opportunities in the agricultural value chain |
| Key observation programs for youth in Agric | Key observations based on experience or opinions of participants related to agricultural programs targeted at youth. |
| Key observation value chain mapping | Key observations based on experience or opinions of participants related to the value chain mapping exercise |
| Key observations farming and Agric careers | Key observations based on experience or opinions of participants related to farming and agriculture careers |
| Knowledge of value-chain activities | What participants know and understand about the agricultural value chain and activities involved in it |
| Least employment opportunities | Participants' opinions about where the 'least employment opportunities' are for young people in the agricultural value chain |
| Most employment opportunities | Participant's opinions on where most employment opportunities are for young people in the agriculture value chain |
| Programmes for youth in Agric | Participant's views on programmes targeted at attracting young people into agriculture. |
| Recommended action for attracting youth | Participant's views and recommendations on what should be done to attract youth into agriculture. |


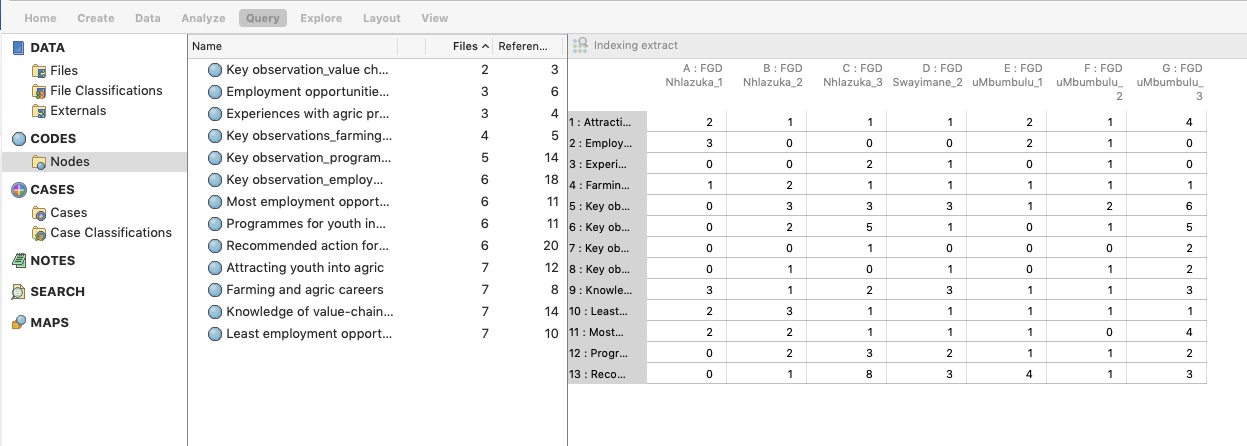


Figure 1: An extract of indexed data from the framework in Nvivo. The rows show the categories from the framework, and focus groups in each community are shown in the columns.
